# Supplementary material for: Characterization of Spatial Air Pollution Patterns Near a Large Railyard Area in Atlanta, Georgia
Source: Int J Environ Res Public Health. 2019 Feb 13;16(4):535. doi: 10.3390/ijerph16040535 (PMC6407103; doi:10.3390/ijerph16040535)
Supplement: Supplementary file 1 [file ijerph-16-00535-s001.pdf]

Supplemental Information

Characterization of Spatial Air Pollution Patterns Near a Large Railyard Area in Atlanta, Georgia

Halley L. Brantley [1], Gayle S.W. Hagler [2,*], Scott C. Herndon [3], Paola Massoli [3], Michael H. Bergin [4] and Armistead G. Russell [5]

[1] Department of Statistics, North Carolina State University, Raleigh, NC 27607, USA
[2] U.S. EPA Office of Research and Development, Research Triangle Park, NC 27711, USA
[3] Aerodyne Research Inc., Billerica, MA 01821, USA
[4] Department of Civil and Environmental Engineering, Duke University, Durham, NC 27708, USA
[5] Civil and Environmental Engineering, Georgia Institute of Technology, Atlanta, GA 30332, USA
* Correspondence: hagler.gayle@epa.gov

Fig S1: Concentrations of BC aggregated by 50 m road segment during (left) winds from the north-northeast (middle) winds from the south-southwest and (right) calm winds. Segments are binned so that each color represents an equal number of road segments. Blue arrows represent range of mean hourly wind directions.

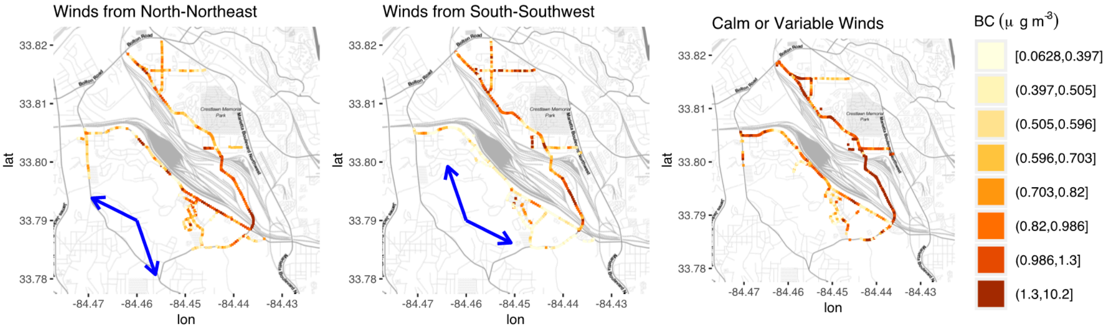

Fig. S2: Concentrations of Benzene aggregated by 50 m road segment during (left) winds from the north-northeast (middle) winds from the south-southwest and (right) calm winds. Segments are binned so that each color represents an equal number of road segments. Blue arrows represent range of mean hourly wind directions.

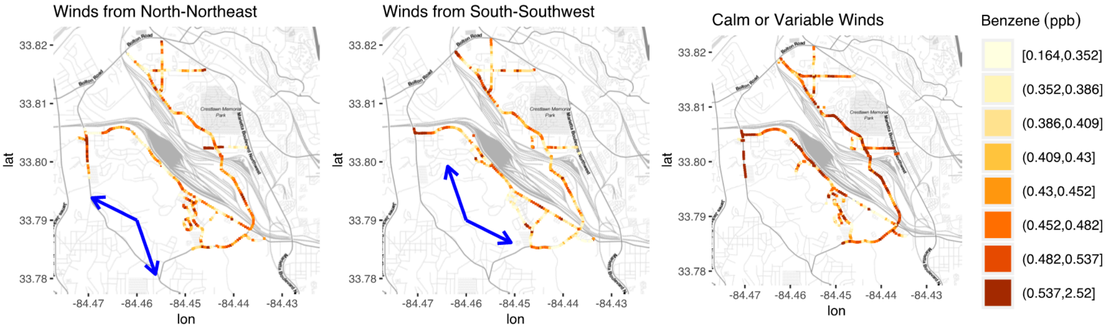

Fig. S3: Concentrations of CO aggregated by 50 m road segment during (left) winds from the north-northeast (middle) winds from the south-southwest and (right) calm winds. Segments are binned so that each color represents an equal number of road segments. Blue arrows represent range of mean hourly wind directions.

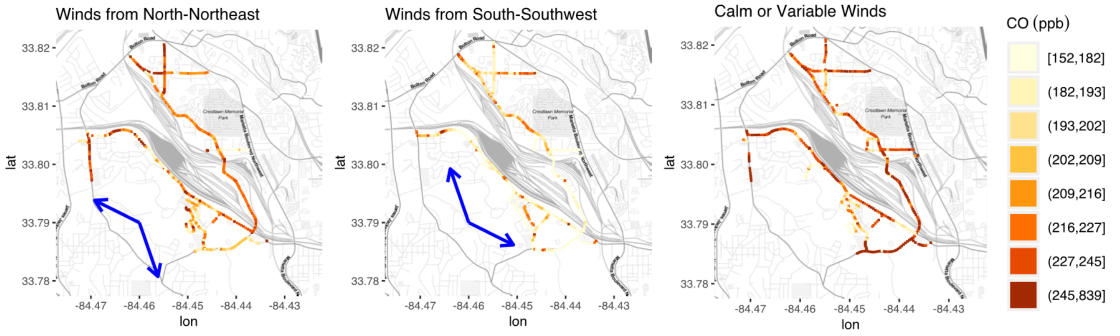

Fig. S4: Concentrations of particle extinction aggregated by 50 m road segment during (left) winds from the north-northeast (middle) winds from the south-southwest and (right) calm winds. Segments are binned so that each color represents an equal number of road segments. Blue arrows represent range of mean hourly wind directions.

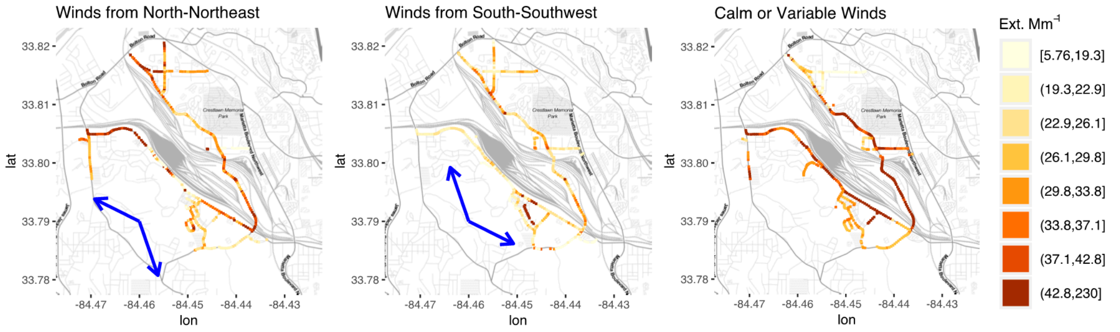

Fig. S5: Concentrations of NO aggregated by 50 m road segment during (left) winds from the north-northeast (middle) winds from the south-southwest and (right) calm winds. Segments are binned so that each color represents an equal number of road segments. Blue arrows represent range of mean hourly wind directions.

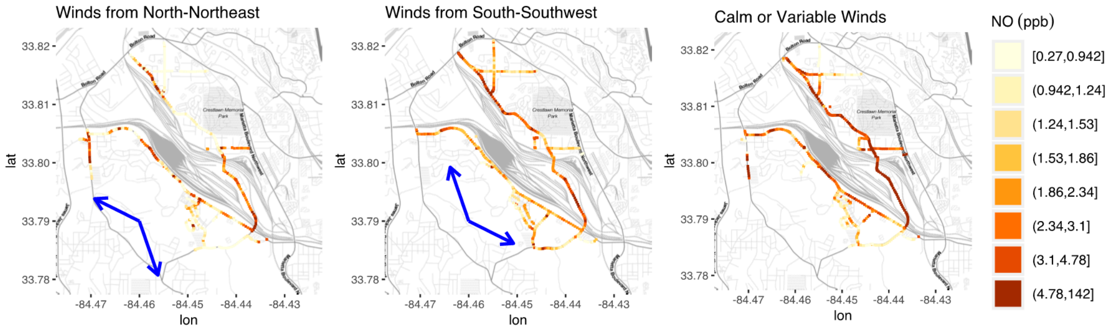

Fig. S6: Concentrations of NOy aggregated by 50 m road segment during (left) winds from the north-northeast (middle) winds from the south-southwest and (right) calm

winds. Segments are binned so that each color represents an equal number of road segments. Blue arrows represent range of mean hourly wind directions.

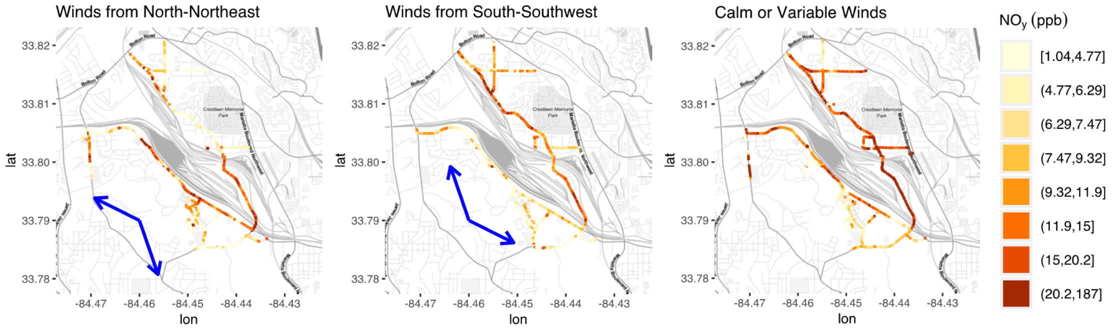

Fig. S7: Particle Number aggregated by 50 m road segment during (left) winds from the north-northeast (middle) winds from the south-southwest and (right) calm winds. Segments are binned so that each color represents an equal number of road segments. Blue arrows represent range of mean hourly wind directions.

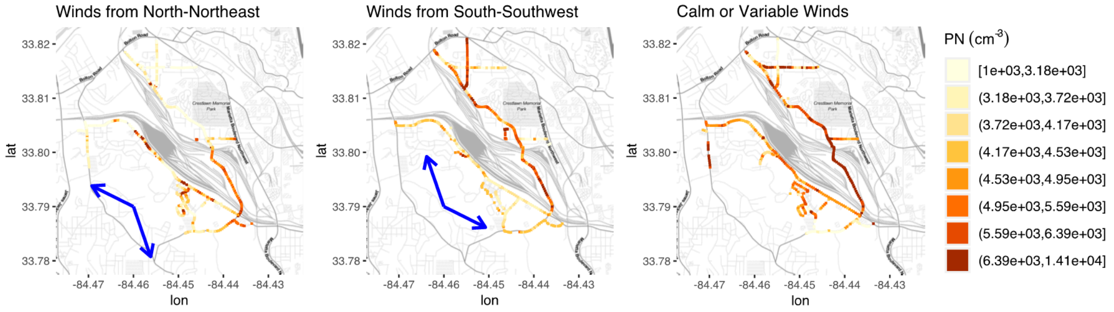

Fig. S8: Concentrations of Toluene aggregated by 50 m road segment during (left) winds from the north-northeast (middle) winds from the south-southwest and (right) calm winds. Segments are binned so that each color represents an equal number of road segments. Blue arrows represent range of mean hourly wind directions.

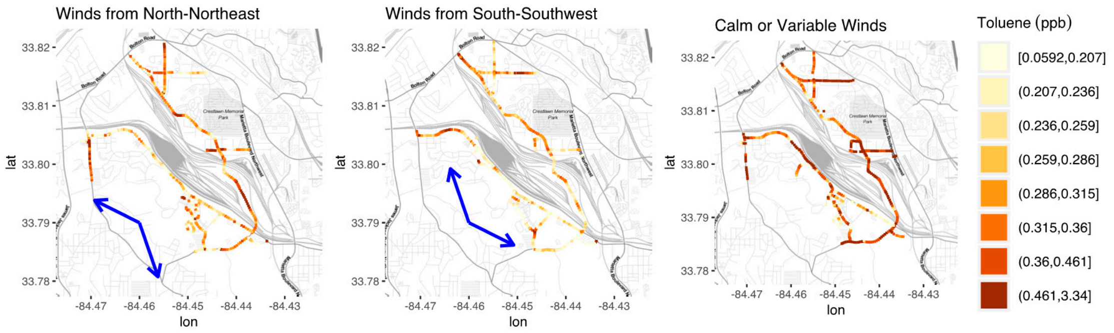

Table S1: Estimated effect of each of the predictors on the mean log transformed pollutant concentration, using both the independent and spatial error models. Effects have been exponentiated to represent factor increases.

	$exp(\beta_0)$ (intercept)		$exp(\beta_1)$(calm)		$exp(\beta_2)$(railyard)		$exp(\beta_3)$(downwind)		$exp(\beta_4)$(distance)	
	Independent	Spatial	Independent	Spatial	Independent	Spatial	Independent	Spatial	Independent	Spatial
NO	1.941	1.024	1.514	1.701	1.941	1.81	1.117	1.453	0.405	0.455
NO2	6.357	1.026	1.59	2.04	1.852	1.946	1.229	1.668	0.472	0.627
NOy	8.67	1.02	1.48	1.714	1.803	1.794	1.183	1.404	0.482	0.581
BC	0.605	1.019	1.422	1.466	1.778	1.804	1.218	1.457	0.673	0.723
CO	200.524	1.005	1.13	1.208	1.019	1.038	1.026	1.025	1.055	1.098
Extinction	25.766	1.014	1.401	1.488	1.236	1.267	1.092	1.172	0.837	0.803
PN	4363.665	1.011	1.164	1.21	1.309	1.27	1.002	1.138	0.743	0.743
Benzene	0.43	1.008	1.135	1.149	1.035	1.01	1.015	0.989	0.939	0.934
Toluene	0.292	1.014	1.352	1.37	1.265	1.126	0.99	0.906	1.126	1.039
Acetal.	2.084	1.005	1.138	1.137	1.106	1.031	1.079	1.048	0.945	0.866
